# Geometric characterization of the persistence of 1D maps

**DOI:** 10.1007/s41468-023-00126-9

**Published:** 2023-06-17

**Authors:** Ranita Biswas, Sebastiano Cultrera di Montesano, Herbert Edelsbrunner, Morteza Saghafian

**Affiliations:** IST Austria (Institute of Science and Technology Austria), Klosterneuburg, Austria

**Keywords:** Geometric networks, 1-Dimensional maps, Extended persistent homology, Variation, Stable marriage, 55N31

## Abstract

We characterize critical points of 1-dimensional maps paired in persistent homology geometrically and this way get elementary proofs of theorems about the symmetry of persistence diagrams and the variation of such maps. In particular, we identify branching points and endpoints of networks as the sole source of asymmetry and relate the cycle basis in persistent homology with a version of the stable marriage problem. Our analysis provides the foundations of fast algorithms for maintaining a collection of sorted lists together with its persistence diagram.

## Introduction

We consider 1-dimensional real-valued maps, by which we mean continuous functions on compact 1-dimensional spaces, such as the the unit circle, the unit interval, and more general geometric networks. Such maps are ubiquitous and arise in developmental biology [e.g. rythmic gene expression (Dequeant et al. 2008)], physiology [e.g. heart-rate (Graff et al. 2021)], and numerous other fields.

Maps on compact 1-dimensional spaces allow for local conditions that characterize features identified by persistent homology, as we will explain in the technical sections of this paper. Indeed, the main contribution of this paper is a local characterization of the pairing of critical points in persistent homology. Let $f :{{\mathbb {G}}}\rightarrow {{\mathbb {R}}}$ be a *generic map on a compact geometric network*, by which we mean that ${{\mathbb {G}}}$ is a bounded geometric realization of a graph, and *f* is continuous with isolated critical points and distinct critical values. We refer to the critical points as *homological minima* and *homological maxima*, which may be minima or maxima in the interior of intervals, or endpoints and branching points of the network. The local characterization of persistent homology is formulated in terms of windows, each the product of a connected subset of ${{\mathbb {G}}}$ and the range of *f* restricted to this subset. Such a product is defined by a pair of critical points, *a*, *b*, and provided it satisfies the conditions detailed in Definitions 3.1, 4.1, 4.3, and 5.3, we refer to it as a *window* denoted ${W}{({a},{b})}$. We distinguish between windows with *simple wave* (see Fig. 2), with *short wave* (Fig. 3), with *branching wave* (Fig. 4), and windows of *component* and of *cycle* (Fig. 5). To state the main theorem, we recall that the *extended persistence diagram* of *f*, denoted $\textsf{Dgm}_{}{({f})}$, consists of three subdiagrams, denoted $\textsf{Ord}_{}{({f})}$, $\textsf{Rel}_{}{({f})}$, and $\textsf{Ess}_{}{({f})}$; see Cohen-Steiner et al. (2009) for details. Whenever necessary, we restrict the diagrams to a given dimension, which we list as a subscript.

### Main Theorem

Let $f :{{\mathbb {G}}}\rightarrow {{\mathbb {R}}}$ be a generic map on a compact geometric network, let *a* be a homological minimum, with $f(a) = A$, and let *b* be a homological maximum, with $f(b) = B$. Then

(i): $(A, B) \in \textsf{Ord}_{0}{({f})}$ iff ${W}{({a},{b})}$ is a window with wave of *f*,
(ii): $(B, A) \in \textsf{Rel}_{1}{({f})}$ iff ${W}{({b},{a})}$ is a window with wave of $-f$,
(iii): $(A, B) \in \textsf{Ess}_{0}{({f})}$ iff ${W}{({a},{b})}$ is a window of component of *f*,
(iv): $(B, A) \in \textsf{Ess}_{1}{({f})}$ iff ${W}{({a},{b})}$ is a window of cycle of *f*.

Compact geometric networks contain the unit circle as a special case. A direct implication of the Main Theorem is that the extended persistent diagram of a function defined on the unit circle is symmetric. This is not the case for more general geometric networks. Indeed, we will see that endpoints and branching points complicate the structure of the windows with wave, causing the persistent pairs of *f* to be different from the ones of $-f$.

Another implication of the Main Theorem is a relation between the variation and the total persistence. The *variation* of a real-valued map quantifies the total amount of local change in the map. According to the Koksma–Hlawka inequality, the error of a numerical integration is bounded from above by the variation of the map times the discrepancy of the points at which the map is evaluated (Hlawka 1961; Koksma 1942). For a 1-dimensional, compact, and piecewise differentiable map, the variation is the integral of the absolute derivative. It is also the *total persistence* of the map, as we will prove in this paper. The variation is thus a numerical summary of the more detailed information expressed in the persistence diagram. Not unlike the Fourier transform, this diagram decomposes the variation into different scales.

### Main Corollary

For a generic map, $f :{{\mathbb {G}}}\rightarrow {{\mathbb {R}}}$, on a compact geometric network, the variation equals the total persistence: $\textrm{Var}{({f})} = {{\Vert {\textsf{Dgm}_{}{({f})}}\Vert }_1}$.

This relation has been known in the special case of a map on the unit circle; see e.g. Dequeant et al. (2008). Beyond this case, the relation is new. The main technical insights needed to prove these results are nesting properties of the windows that characterize persistence pairs. Indeed, the projections of any two windows onto the geometric network are either nested or disjoint and thus form the basis of a topology of the network.

**Outline.** Sect. 2 introduces basic terminology and properties of maps, homology, and persistent homology. Section 3 studies maps on the unit circle. Section 4 considers maps on the unit interval and on geometric trees. Section 5 extends the results to maps on geometric networks. Section 6 concludes the paper.

## Background

This paper deals exclusively with continuous real-valued maps on 1-dimensional compact spaces. We therefore need only a few mathematical prerequisites and we recommend (Edelsbrunner and Harer 2010) for a more comprehensive introduction to persistent homology. Throughout this section, we first introduce the relevant terminology for the circle case and then mention how it extends to compact 1-dimensional spaces.

### Maps and spaces

**Fig. 1:**
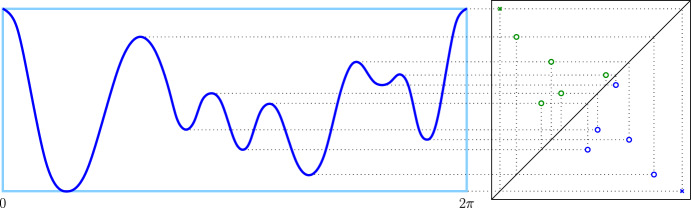
*Left:* the graph of a generic function on the circle with the global maximum at $0 = 2\pi$. The six minima alternate with the six maxima. *Right:* the persistence diagram of the map. The two points that correspond to the global min-max pair are marked by crosses, while all other points are marked by small circles

Let $f :{{\mathbb {S}}}^1 \rightarrow {{\mathbb {R}}}$ be a continuous map on the unit circle; see Fig. 1. We call *f* *generic* if there is no non-empty open interval along which *f* is constant, so all critical points are isolated, and the values of these critical points are distinct. A *minimum* of such a map is a point $a \in {{\mathbb {S}}}^1$ for which there exists a neighborhood, $N(a) \subseteq {{\mathbb {S}}}^1$, such that $f(a) < f(x)$ for all $x \in N(a)$. Symmetrically, a *maximum* is a point $a \in {{\mathbb {S}}}^1$ such that $f(a) > f(x)$ for all $x \in N(a)$. The minima and maxima alternate in a trip around the circle, which implies that there are equally many of them. There is exactly one *global minimum*, $a_0$, and one *global maximum*, $b_0$, which satisfy $f(a_0) \le f(x) \le f(b_0)$ for all $x \in {{\mathbb {S}}}^1$. We note that the stability of the persistence diagram (Cohen-Steiner et al. 2007) makes the assumption of distinct critical values unnecessary, but we include it in the definition of genericity to simplify arguments at many places.

By a *geometric network* we mean the realization of an abstract graph in some Euclidean space: each vertex is mapped to a point, and each edge to a curve connecting the images of its vertices. We are not concerned with the details of the embedding, except that different vertices map to different points, and curves do not intersect except possibly at shared endpoints. For convenience, we restrict ourselves to finite graphs in which every vertex has degree 1 or 3. The constraint on the vertex degrees is not really a limitation since we can replace a degree-*k* vertex by a tree with $k-2$ vertices, all of degree 3, and if the edges in the tree approach zero length, we can recover the original topology in the limit. Similar substitutions can be used to model multi-edges and cycles. If a geometric network is connected and without cycle, we call it a *geometric tree*.

Besides minima and maxima, geometric networks contain two other types of critical points that play a central role in our analysis: the *endpoints* (degree-1 vertices) and *branching points* (degree-3 vertices). An endpoint has $\searrow$-*type* or $\nearrow$-*type* if its value is larger or smaller than the values of the points in a sufficiently small neighborhood, respectively. Similarly, we allow for two types of degree-3 vertices: y-*type* (one $\searrow$- and two $\nearrow$-type vertices glued to each other) and $\lambda$-*type* (one $\nearrow$- and two $\searrow$-type vertices glued to each other). We call the function $f :{{\mathbb {G}}}\rightarrow {{\mathbb {R}}}$ *generic* if all critical points are isolated and their values are distinct. A *critical value* of *f* is the value of a critical point; all other values are *non-critical*.

Call $f :{{\mathbb {G}}}\rightarrow {{\mathbb {R}}}$ *piecewise differentiable* if there is a decomposition of ${{\mathbb {G}}}$ into a finite number of curves such that the restriction of *f* to the interior of each curve is differentiable. For example, if ${{\mathbb {G}}}$ is a straight-line embedding of a finite graph, and *f* is the linear extension of its values at the vertices, then *f* is piecewise linear and therefore also piecewise differentiable. We define the *variation* of a piecewise differential map as the integral, over all interior points of the curves in the decomposition, of the absolute value of the derivative at these points.

### Homology with ${{\mathbb {Z}}}/2{{\mathbb {Z}}}$ coefficients

To keep the algebraic discussion of homology as elementary as possible, we use modulo-2 arithmetic; that is: we construct homology groups with ${{\mathbb {Z}}}/2{{\mathbb {Z}}}$ coefficients. For compact 1-dimensional spaces, such groups are straightforward objects, so we can side-step the formal introduction of homology. For a more comprehensive treatment, we recommend standard texts in algebraic topology, for example Hatcher (Hatcher 2002).

Given a map, $f :{{\mathbb {S}}}^1 \rightarrow {{\mathbb {R}}}$, the *sublevel set* at $t \in {{\mathbb {R}}}$ is $f_t = f^{-1} (-\infty , t]$, and the *superlevel set* is $f^t = f^{-1} [t, \infty )$. Let $A_0$ and $B_0$ be the values of the global minimum and the global maximum, respectively. For a non-critical value, *t*, we have the following three cases:

- $$t < A_0$$: $$f_t = \emptyset $$ and $$f^t = {{\mathbb {S}}}^1$$;
- $A_0< t < B_0$: $f_t$ consists of a positive number of connected components, each a closed arc with non-empty interior, and $f^t$ consists of the same number of connected components of the same type;
- $$t > B_0$$: $$f_t = {{\mathbb {S}}}^1$$ and $$f^t = \emptyset $$.

We use *homology* to formally distinguish between these cases. In particular, the rank of $\textsf{H}_0 (f_t)$ is the number of connected components of the sublevel set, and the rank of $\textsf{H}_1 (f_t)$ is the number of cycles. Note that we have no cycle for $t < B_0$ and one cycle for $t > B_0$. Compare this with the *homology* of ${{\mathbb {S}}}^1$ *relative* to $f^t$, denoted $\textsf{H}_i ({{\mathbb {S}}}^1, f^t)$, where we have ${\mathrm{rank\,}{\textsf{H}_0({{\mathbb {S}}}^1, f^t)}} = {\mathrm{rank\,}{\textsf{H}_1 (t_t)}} = 0$ for $t < B_0$ and ${\mathrm{rank\,}{\textsf{H}_0({{\mathbb {S}}}^1, f^t)}} = {\mathrm{rank\,}{\textsf{H}_1 (f_t)}} = 1$ for $t > B_0$. More interesting is the case $i=1$, for which the relative homology group counts the open arcs in ${{\mathbb {S}}}^1 {\setminus } f^t$. By Lefschetz duality, the (absolute) homology groups and the relative homology groups are isomorphic: $\textsf{H}_i (f_t) \simeq \textsf{H}_{1-i} ({{\mathbb {S}}}^1, f^t)$, for $i=0,1$ and for all non-critical values, *t* of *f*. This is an elementary insight for the circle and is also true for higher-dimensional manifolds. It does not hold for more general spaces, not even for the unit interval. On the other hand, both homology and relative homology generalize and can be used to count connected components and cycles in geometric networks and the sub- and superlevel sets of maps on them.

### Persistent homology

Persistent homology arises when we keep track of sub- and superlevel sets while *t* changes continuously. We again take advantage of the relative simplicity provided by the restriction to compact 1-dimensional spaces and avoid the introduction of the concept in full generality. For more comprehensive background, we refer to Edelsbrunner and Harer (2010). Specifically, we use the framework that is referred to as *extended persistent homology*, which is constructed in two phases, first growing the sublevel set until it exhausts the space, and second doing the same with the superlevel set. We explain this for a generic map on the unit circle.

In *Phase One*, we increase *t* from $-\infty$ to $\infty$ and use $\textsf{H}_0 (f_t)$ and $\textsf{H}_1 (f_t)$ to do the book-keeping. A connected component is *born* when *t* passes the value of a minimum, and the component *dies* merging into another, older component when *t* passes the value of a maximum. There is one exception: when *t* passes $B_0$, then no component dies and instead a cycle is born. We pair up the minimum, *a*, and the maximum, *b*, responsible for the birth and death of a component and represent the two events by the point (*f*(*a*), *f*(*b*)) in the plane.

In *Phase Two*, we decrease *t* from $\infty$ to $- \infty$ and use $\textsf{H}_0 ({{\mathbb {S}}}^1, f^t)$ and $\textsf{H}_1 ({{\mathbb {S}}}^1, f^t)$ to do the book-keeping. We enter Phase Two with a component born at $A_0 = f(a_0)$ and a cycle born at $B_0 = f(b_0)$, both of which did not yet die. The component dies in relative homology right at the beginning of Phase Two, when *t* passes $B_0$ (from the top going down), while the cycle lasts until the end, and dies when *t* passes $A_0$. This gives two pairs represented by the points $(A_0, B_0)$ and $(B_0, A_0)$. During Phase Two, a (relative 1-dimensional) cycle is born when *t* passes the value of a (non-global) maximum, and this cycle dies when *t* passes the value of a (non-global) minimum. Like in Phase One, we pair up the maximum, *b*, with the minimum, *a*, responsible for the birth and death of the cycle and represent the two events by the point (*f*(*b*), *f*(*a*)) in the plane.

### Persistence diagrams

The events during the two phases are recorded in the *persistence diagram* of *f*, denoted $\textsf{Dgm}_{}{({f})}$, which is a multi-set of points, each marking the birth and death of a component or cycle; see Fig. 1. We distinguish between three disjoint subdiagrams, $\textsf{Dgm}_{}{({f})} = \textsf{Ord}_{}{({f})} \sqcup \textsf{Rel}_{}{({f})} \sqcup \textsf{Ess}_{}{({f})}$, in which the *ordinary subdiagram* records the pairs in Phase One, the *relative subdiagram* records the pairs in Phase Two, and the *essential subdiagram* records the pairs that straddle the two phases. Whenever convenient, we list the dimension as a subscript, writing $\textsf{Dgm}_{i}{({f})}$ for the points that represent *i*-dimensional homology classes, and similarly for the subdiagrams. For a 1-dimensional map, we have $\textsf{Ord}_{}{({f})} = \textsf{Ord}_{0}{({f})}$, $\textsf{Rel}_{}{({f})} = \textsf{Rel}_{1}{({f})}$, but $\textsf{Ess}_{}{({f})} = \textsf{Ess}_{0}{({f})} \sqcup \textsf{Ess}_{1}{({f})}$. Recall that for a map on the unit circle, Lefschetz duality implies that the pairs in Phase One are the same as in Phase Two, only reversed. Similarly, for every pair straddling the two phases, there is also the reversed pair straddling the two phases. This implies that $\textsf{Dgm}_{}{({f})}$ is symmetric across the main diagonal, with the caveat that a point $(f(a), f(b)) \in \textsf{Dgm}_{i}{({f})}$ maps to the point $(f(b), f(a)) \in \textsf{Dgm}_{1-i}{({f})}$; see Fig. 1 and Cohen-Steiner et al. (2009) for details. This property no longer holds for maps on non-manifold spaces, such as the unit interval, geometric trees, and general geometric networks. Nevertheless, the persistence diagram and its subdiagrams are useful book-keeping tools for the homology of the sub- and superlevel sets of maps on such more general spaces. Specifically, the ordinary subdiagram records the components of the sublevel set that are born and die during Phase One. The essential subdiagram records the homology of the geometric network, since its classes are born but do not die during Phase One. Finally, the relative subdiagram records the relative cycles in the network modulo the superlevel set.

For a point $(A,B) \in \textsf{Dgm}_{}{({f})}$, we think of $|B-A|$ as the life-time or *persistence* of the corresponding component or cycle. Taking the sum, over all points in the multi-set, we get what we call the *total persistence* of *f*:

1 $$\begin{aligned} {{\Vert {\textsf{Dgm}_{}{({f})}}\Vert }_1} = \sum \nolimits _{(A,B) \in \textsf{Dgm}_{}{({f})}} |B-A| . \end{aligned}$$

For a map on the unit circle, the global minimum and the global maximum contribute $2 |B_0 - A_0|$ to this measure. Everything beyond that is due to wrinkles in the map and may be regarded as a measure of how interesting or noisy the map is.

An important property of persistence diagrams is their stability, which was first proved in Cohen-Steiner et al. (2007). Assuming *f* and *g* are generic maps on the same compact geometric network, this theorem asserts that the bottleneck distance between $\textsf{Dgm}_{}{({f})}$ and $\textsf{Dgm}_{}{({g})}$ is bounded from above by ${\Vert {f}-{g}\Vert }_{\infty }$. It follows that every continuous map has a generic perturbation whose total persistence is arbitrarily close to that of the original map. It also implies that the restriction to maps whose critical points have distinct values is unnecessary while convenient.

## The circle case

We treat the circle separately and before considering more general geometric networks because it is the only connected 1-manifold among them.

### Maps on the circle

We consider generic maps on the unit circle and introduce the notion of a window to characterize the critical points paired by persistent homology. After establishing this connection, we get elementary proofs of fundamental properties of maps on the circle.

**Fig. 2:**
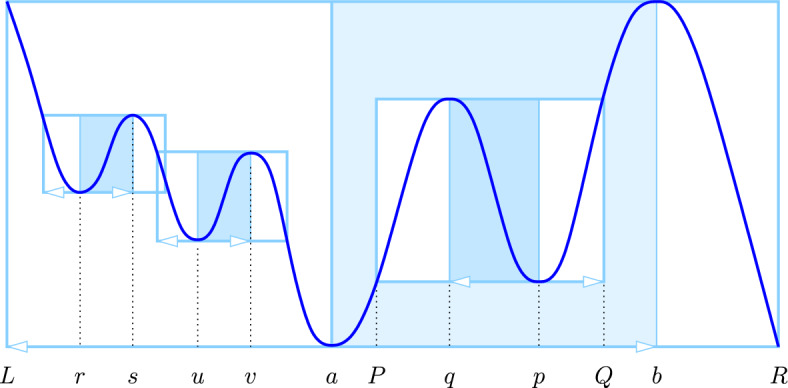
The triple-panel window with simple wave spanned by *a* and *b*. There are two children in the in-panel, spanned by *r*, *s* and *u*, *v*, there is one child in mid-panel, spanned by *q*, *p*, and there is no child in the out-panel. The triple-panel windows spanned by *r*, *s* and *u*, *v* overlap, while the corresponding double-panel windows are disjoint

Let *a* be a minimum and *b* a maximum of a generic map, $f :{{\mathbb {S}}}^1 \rightarrow {{\mathbb {R}}}$, write $A = f(a)$, $B = f(b)$, and let $J = {J}{({a},{b})}$ be the connected component of $f^{-1} [A, B]$ that contains both *a* and *b*. It may be a closed interval, the entire circle, or empty if no such component exists. We call ${W}{({a},{b})} = J \times [A, B]$ the *frame* with *support* *J* *spanned* by *a* and *b*, and we say ${W}{({a},{b})}$ *covers* the points $x \in J$. When *J* is an interval, *a* and *b* decompose it into three (closed) subintervals, which we read in a direction so that *a* precedes *b*: ${J_\textrm{in}}$ before *a*, ${J_\textrm{mid}}$ between *a* and *b*, and ${J_\textrm{out}}$ after *b*. Correspondingly, we call ${J_\textrm{in}}\times [A,B]$, ${J_\textrm{mid}}\times [A,B]$, and ${J_\textrm{out}}\times [A,B]$ the *in-*, *mid-*, and *out-panels* of ${W}{({a},{b})}$. We orient the in- and mid-panels away from the minimum, while we leave the out-panel without orientation; see Fig. 2.

#### Definition 3.1

(Windows for Circles) Let *a* be a (non-global) minimum and *b* a (non-global) maximum, and assume that ${J}{({a},{b})}$ is non-empty. We call ${W}{({a},{b})}$, a *triple-panel window with simple wave* if the values at the endpoints of $J_\textrm{in}, J_\textrm{mid}, J_\textrm{out}$ are *B*, *A*, *B*, *A* in this sequence.

We will sometimes consider a *double-panel window*, which consists of the in-panel and the mid-panel. It contains the graph of the component in the sublevel set that grows from the minimum until it merges with another component at the corresponding maximum. We show that the windows with wave characterize the paired critical points, while noting that the global min-max pair is special and not subject to the following claim.

#### Theorem 3.2

(Characterization for Circles) Let $f :{{\mathbb {S}}}^1 \rightarrow {{\mathbb {R}}}$ be generic, let *a* be a (non-global) minimum with $f(a) = A$, and *b* a (non-global) maximum with $f(b) = B$. Then (*A*, *B*) and (*B*, *A*) are points in the ordinary and relative subdiagrams of $\textsf{Dgm}_{}{({f})}$ iff the frame spanned by *a* and *b* is a triple-panel window with simple wave.

#### Proof

“$\Longleftarrow$”. Let *a*, *b* span ${W}{({a},{b})} = [L,R] \times [A, B]$, and assume that *a* is to the left of *b*, as in Fig. 2. Consider the component of $f_t$ that contains *a* as *t* increases from $- \infty$ to $\infty$. This component is born at $t = A$. Since $A \le f(x) \le B$ for all $L \le x \le b$, the component grows—occasionally by incorporating other, younger components—but never dies before *t* reaches *B*. At $t = B$, the component meets another component at *b*, and since ${W}{({a},{b})}$ is a triple-panel window with wave, this other component is older. It follows that *a*, *b* are paired.

“$\Longrightarrow$”. We suppose that *a*, *b* are paired. In other words, a component of $f_t$ is born at $t = A$, and *a* remains the point with minimum value in this component until $t = B$, when the component merges with another, older component. Let [*L*, *b*] and [*b*, *X*] be the components right before merging. The graph of *f* restricted to [*L*, *b*] describes the history of the component born at $t = A$, which implies that it is contained in $[L,b] \times [A, B]$. The other component is born earlier, so [*b*, *X*] contains points that have the same value as *a*. Let *R* be the leftmost such point. By construction, the graph of *f* restricted to [*L*, *R*] is contained in $[L,R] \times [A, B]$, which implies that ${W}{({a},{b})}$ is a triple-panel window with simple wave. $\square$

In addition to the points in the ordinary and relative subdiagrams—which are characterized by Theorem 3.2—$\textsf{Dgm}_{}{({f})}$ contains two more points, namely $(A_0, B_0)$ and $(B_0, A_0)$ in the essential subdiagram. With $A_0 < B_0$ the values of the global minimum and the global maximum, the first point represents the component and the second the cycle of the circle. There is no ambiguity which critical points of *f* are paired in persistent homology. Theorem 3.2 thus implies that for every minimum there is a unique maximum such that the corresponding frame is a window.

### Nesting of windows

As illustrated in Fig. 2, two windows can be *nested* (one is a subset of the other), they can be *disjoint*, and they can *overlap*. We will see that any overlap is limited. We call ${W}{({u},{v})}$ a *child* of ${W}{({a},{b})}$, and ${W}{({a},{b})}$ a *parent* of ${W}{({u},{v})}$, if ${W}{({u},{v})}$ is nested inside one of the panels of ${W}{({a},{b})}$, and there is no other window nested between the two.

#### Lemma 3.3

(Nesting in Circle) Let $f :{{\mathbb {S}}}^1 \rightarrow {{\mathbb {R}}}$ be generic, and let ${W}{({a},{b})}$ be a triple-panel window with simple wave and supports ${J_\textrm{in}}, {J_\textrm{mid}}, {J_\textrm{out}}$ of its panels. If ${W}{({u},{v})}$ is another triple-panel window and $u \in {J_\textrm{in}}, {J_\textrm{mid}}, {J_\textrm{out}}$, then ${W}{({u},{v})}$ is nested inside the corresponding panel of ${W}{({a},{b})}$.

#### Proof

We first consider the mid-panel of ${W}{({a},{b})}$, which we assume is oriented from left to right, so $a < b$. Moving from $x=a$ to $x=b$, we encounter an alternating sequence of minima and maxima, starting with *a* and ending with *b*. If *a* and *b* are the only critical points in this sequence, then the statement is vacuously true. Otherwise, let $a< p < b$ be the minimum with the smallest value, *f*(*p*). There is at least one maximum to its left, and we let $a< q < p$ be the maximum with the largest value, *f*(*q*); see Fig. 2. Drawing a horizontal line from (*p*, *f*(*p*)) to the left, we intersect the graph of *f* in (*P*, *f*(*p*)), and drawing a horizontal line from (*q*, *f*(*q*)) to the right, we intersect the graph in (*Q*, *f*(*q*)). By construction, $a< P< q< p< Q < b$ as well as $f(p) \le f(x) \le f(q)$ for all $P \le x \le Q$. Hence, ${W}{({p},{q})}$ is a triple-panel window with simple wave nested inside the mid-panel of ${W}{({a},{b})}$.

To continue, we subdivide [*a*, *b*] at *q* and *p*, and apply the same argument in each of the three sections to get a pairing of all critical points in the interior of [*a*, *b*]. Their frames are therefore triple-panel windows with simple waves nested inside mid-panel of ${W}{({a},{b})}$. Repeating the argument for the in-panel and the out-panel, we obtain the desired claim.

Recall that a double-panel window is obtained by dropping the out-panel. The double-panel windows can be nested or disjoint, but in contrast to the triple-panel windows, they cannot overlap. Indeed by Lemma 3.3, non-nested windows do not cover each other’s critical points. It follows that the overlap is limited to the in-panel of one and the out-panel of the other window. Since we drop the out-panel, double-panel windows cannot overlap.

### Consequences: symmetry and variation

We use the hierarchies of triple- and double-panel windows to prove two folklore results about real-valued maps on the circle. The first is a statement of symmetry that follows from Alexander duality. Given a multiset of points in ${{\mathbb {R}}}^2$, such as $\textsf{Dgm}_{}{({f})}$, we write $\textsf{Dgm}^\circ _{}{({f})}$ for the central reflection, which negates coordinates. Similarly, we write $\textsf{Dgm}^R_{}{({f})}$ for the reflection across the major diagonal, which switches coordinates, and $\textsf{Dgm}^r_{}{({f})}$ for the reflection across the minor diagonal, which negates and switches coordinates.

#### Corollary 3.4

(Strong Symmetry for Circles) Let $f :{{\mathbb {S}}}^1 \rightarrow {{\mathbb {R}}}$ be generic. Then $\textsf{Dgm}_{}{({f})} =\textsf{Dgm}^R_{}{({f})}$ and $\textsf{Dgm}_{}{({-f})} = \textsf{Dgm}^r_{}{({f})}$.

#### Proof

A window with simple wave of *f* is also such a window of $-f$. Hence, $(A,B) \in \textsf{Ord}_{}{({f})}$ iff $(B,A) \in \textsf{Rel}_{}{({f})}$. Recall also that $\textsf{Ess}_{}{({f})}$ consists of two points, $(A_0, B_0)$ and $(B_0, A_0)$, in which $A_0 = \min _x f(x)$ and $B_0 = \max _x f(x)$. This implies $\textsf{Dgm}_{}{({f})} = \textsf{Dgm}^R_{}{({f})}$.

To relate *f* with $-f$, note that both have the same critical points, except that minima switch with maxima. Since ${W}{({a},{b})} = J \times [A,B]$ is a triple-panel window of *f* iff ${W}{({b},{a})} = J \times [-B,-A]$ is a triple-panel window of $-f$, this implies that we get the diagram of $-f$ by negating and switching the coordinates; that is: $\textsf{Dgm}_{}{({-f})} = \textsf{Dgm}^r_{}{({f})}$. $\square$

To state the second result, we recall that the *variation* of a 1-dimensional function is the total amount of climbing up and down. We claim that for $f :{{\mathbb {S}}}^1 \rightarrow {{\mathbb {R}}}$, this is the total persistence of *f*, which we recall is the sum of $|B-A|$ over all points $(A,B) \in \textsf{Dgm}_{}{({f})}$.

#### Corollary 3.5

(Variation for Circles) Let $f :{{\mathbb {S}}}^1 \rightarrow {{\mathbb {R}}}$ be generic. Then the variation equals the total persistence of *f*: $\textrm{Var}{({f})} ={{\Vert {\textsf{Dgm}_{}{({f})}}\Vert }_1}$.

#### Proof

We use induction, considering the double-panel windows defined by min-max pairs of *f* in a sequence in which the children precede their parents. Observe that *f* restricted to the support of a double-panel window without children consists of two monotonic pieces. Its contribution to the variation of *f* is twice the height of the window, and so is its contribution to the total persistence. Indeed, the min-max pair corresponds to a point each in the ordinary and the relative subdiagrams, or it corresponds to two points in the essential subdiagram. After recording these contributions, we locally flatten *f* to remove the double-panel window and continue the inductive argument. $\square$

The relation between the variation and the total persistence of a map on ${{\mathbb {S}}}^1$ expressed in Corollary 3.5 was known before. For example, it is used to measure to what extent a noisy cyclic map is periodic (Dequeant et al. 2008). Its generalization to maps on geometric networks stated in Corollary 5.6 is however new.

## The geometric tree case

In this section, we consider networks without cycles, which if connected are trees. We begin with a single edge and continue with geometric trees whose interior vertices have degree 3.

### Maps on the interval

The simplest compact 1-dimensional space that is not a 1-manifold is a line segment, which we refer to as an *interval* and parametrize from 0 to 1. Recall that a map $f :[0,1] \rightarrow {{\mathbb {R}}}$ is *generic* if the minima, maxima, and endpoints are isolated and their values are distinct. Theorem 3.2 applies in the interior of the interval, but we need new kinds of windows that cover the endpoints. Let *a* be a minimum or $\nearrow$-type endpoint and *b* a maximum or $\searrow$-type endpoint of *f*, write $A = f(a)$ and $B = f(b)$, and recall that $J = {J}{({a},{b})}$ is the component of $f^{-1} [A,B]$ that contains both *a* and *b*, with $J = \emptyset$ if no such component exists.

#### Definition 4.1

(Windows for Intervals) Let *a* be a (non-global) minimum or $\nearrow$-type endpoint, and *b* a (non-global) maximum. We call the non-empty frame, ${W}{({a},{b})} = J \times [A,B]$, a *triple-panel window with short wave* if its in-, mid-, out-panels are delimited by $0 \le a< b< x < 1$ or by $1 \ge a> b> x > 0$ such that $f(x) = A$.

**Fig. 3:**
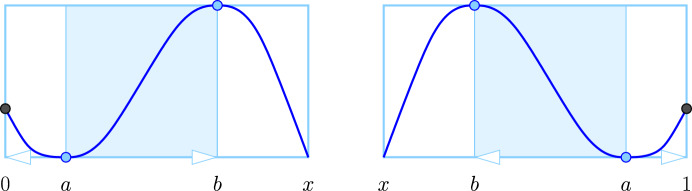
Two triple-panel windows with short wave, oriented from left to right on the *left* and from right to left on the *right*. Both cases may degenerate to zero-width in-panels. The *black* points correspond to endpoints of the interval. There are different ways how a frame can fail to be a window, one being that $f(x) > f(a)$

Observe that Definition 4.1 allows for the cases $a = 0$ and $a = 1$. As illustrated in Fig. 3, a window with short wave covers exactly one endpoint of the interval, and this endpoint is either *a* or a maximum. The case in which the window covers both endpoints is also possible but different and introduced in Definition 5.3. In contrast to windows with simple wave, windows with short wave do not come in symmetric pairs; that is: if ${W}{({a},{b})}$ is a window with short wave of *f*, then ${W}{({b},{a})}$ is not a window with short wave of $- f$.

Because of the asymmetry of windows with short wave, the extension of Theorem 3.2 to intervals requires a separate treatment of the ordinary and relative subdiagrams of $\textsf{Dgm}_{}{({f})}$.

#### Theorem 4.2

(Characterization for Intervals) Let $f :[0,1] \rightarrow {{\mathbb {R}}}$ be generic, let *a* be a minimum or $\nearrow$-type endpoint, with $f(a) = A$, and let *b* be a maximum or $\searrow$-type endpoint, with $f(b) = B$. Then

(i): $(A, B) \in \textsf{Ord}_{}{({f})}$ iff ${W}{({a},{b})}$ is a triple-panel window with simple or short wave of *f*,
(ii): $(B, A) \in \textsf{Rel}_{}{({f})}$ iff ${W}{({b},{a})}$ is a triple-panel window with simple or short wave of $- f$.

#### Proof

The pairs in (i) correspond to components of the sublevel set, which are counted by $\textsf{H}_0$, while the points in (ii) correspond to relative cycles, which are counted by $\textsf{H}_1$. The proof of (i) is almost verbatim the same as that of Theorem 3.2, and we omit the details.

Write ${{\mathbb {I}}}= [0,1]$ and recall that $f^t = f^{-1} [t, 1]$. To prove (ii), we explain the details of how $\textsf{H}_0 (f^t)$ and $\textsf{H}_1 ({{\mathbb {I}}}, f^t)$ are related. To this end, we decrease *t* from $\infty$ to $- \infty$ and show that the two groups change their ranks in parallel, with only one exception at $t = B_0$, the value of the global maximum, when $\textsf{H}_0 (f^t)$ goes from rank 0 to 1 while $\textsf{H}_1 ({{\mathbb {I}}}, f^t)$ remains at rank 0. For this purpose, we consider the long exact sequence of the pair $({{\mathbb {I}}}, f^t)$. We recall that *exactness* means that the image of a map is the kernel of the next map in order along the sequence; see (Edelsbrunner and Harer 2010, Section IV.4) or (Hatcher 2002, Section 2.1) for details. In the 1-dimensional case, all homology groups of dimension other than 0 and 1 are trivial, so the long exact sequence is rather short:

2 $$\begin{aligned} 0 \rightarrow \textsf{H}_1(f^t) \rightarrow \textsf{H}_1({{\mathbb {I}}}) \rightarrow \textsf{H}_1({{\mathbb {I}}},f^t) \rightarrow \textsf{H}_0(f^t) \rightarrow \textsf{H}_0({{\mathbb {I}}}) \rightarrow \textsf{H}_0({{\mathbb {I}}},f^t) \rightarrow 0 . \end{aligned}$$

We have ${\mathrm{rank\,}{\textsf{H}_0 ({{\mathbb {I}}})}} = 1$ and ${\mathrm{rank\,}{\textsf{H}_1 ({{\mathbb {I}}})}} = {\mathrm{rank\,}{\textsf{H}_1 (f^t)}} = 0$ for every *t*. There are only four possibly non-trivial groups, which we related to each other in a case analysis.

- For $t > B_0$, the only non-trivial groups are $\textsf{H}_0 ({{\mathbb {I}}})$ and $\textsf{H}_0 ({{\mathbb {I}}}, \emptyset )$, which both have rank 1. In particular, $\textsf{H}_1 ({{\mathbb {I}}}, f^t)$ and $\textsf{H}_0 (f^t)$ are both trivial and therefore isomorphic.
- For $t \le B_0$, $\textsf{H}_0 ({{\mathbb {I}}}, f^t)$ is trivial, so by the exactness of (2), ${\mathrm{rank\,}{\textsf{H}_1 ({{\mathbb {I}}}, f^t)}} = {\mathrm{rank\,}{\textsf{H}_0 (f^t)}} - 1$.

To finish the argument, we remove the class born at $t = B_0$ from all groups $\textsf{H}_0 (f^t)$ to get two isomorphic persistence modules. It follows that the implied pairing of the critical values is the same, whether we track the components of $f^t$ or the relative cycles of $({{\mathbb {I}}}, f^t)$. Claim (ii) thus follows from (i). $\square$

In addition to the points in the ordinary and relative subdiagrams—which are characterized by Theorem 4.2—$\textsf{Dgm}_{}{({f})}$ contains one more point, namely $(A_0, B_0)$ in the essential subdiagram. This point will be discussed in Sect. 5.

### Maps on geometric trees

If we glue intervals at their endpoints without forming a cycle in the process, we get a *geometric tree*, ${{\mathbb {A}}}= (V, E)$, with *vertices*, *V*, and *edges*, *E*. We restrict ourselves to *degree*-3 *trees*, in which each vertex is an endpoint of either one or three edges. We call $f :{{\mathbb {A}}}\rightarrow {{\mathbb {R}}}$ *generic* if all critical points are isolated, they have distict values, and every degree-3 vertex is either a *y*-type or a $\lambda$-type vertex. It is tempting to consider $\nearrow$- and y-type vertices as minima and $\searrow$- and $\lambda$-type vertices as maxima, but note that components of sublevel sets are born at $\nearrow$-type but not at y-type vertices, and they die at $\lambda$-type but not at $\searrow$-type vertices.

Geometric trees introduce the topological phenomenon of branching, which requires yet another extension of the notion of window with wave. Let *a* be a minimum or $\nearrow$-type vertex, with $f(a) = A$, and *b* a maximum or $\lambda$-type vertex, with $f(b) = B$. Recall that $J = {J}{({a},{b})}$ is the component of $f^{-1} [A,B]$ that contains both *a* and *b*, which is a geometric tree, and that *a*, *b* subdivide *J* into subtrees ${J_\textrm{in}}, {J_\textrm{mid}}, {J_\textrm{out}}$.

#### Definition 4.3

(Windows for Geometric Trees) We call a non-empty frame, ${W}{({a},{b})} = J \times [A,B]$, a *triple-panel window with branching wave* if $f(x) > A$ for every point $x \ne a$ in ${J_\textrm{in}}\cup {J_\textrm{mid}}$, and $f(y) = A$ for at least one point $y \ne b$ in $J_\textrm{out}$.

**Fig. 4:**
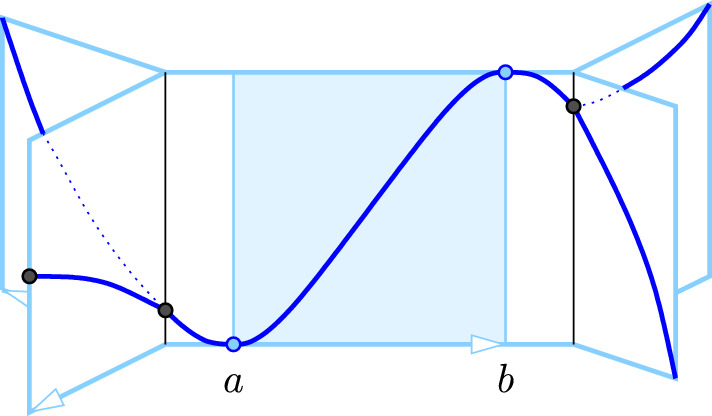
A triple-panel window with branching wave, ${W}{({a},{b})}$. There is a branching point in the in-panel on the *left* and another in the out-panel on the *right*. Branching points and endpoints are marked in *black*. Note that ${W}{({b},{a})}$ violates the conditions in Definition 4.3 for the negated map

Note that the triple-panel windows with simple and short wave satisfy the conditions of Definition 4.3, but there are also others, as illustrated in Fig. 4. We can now generalize Theorem 4.2 from intervals to geometric trees.

#### Theorem 4.4

(Characterization for Geometric Trees) Let $f :{{\mathbb {A}}}\rightarrow {{\mathbb {R}}}$ be a generic map on a compact geometric degree-3 tree, let *a* be a minimum, $\nearrow$-type, or y-type vertex, with $f(a) = A$, and let *b* be a maximum, $\lambda$-type, or $\searrow$-type vertex, with $f(b) = B$. Then

(i): $(A, B) \in \textsf{Ord}_{}{({f})}$ iff ${W}{({a},{b})}$ is a triple-panel window with branching wave of *f*,
(ii): $(B, A) \in \textsf{Rel}_{}{({f})}$ iff ${W}{({b},{a})}$ is a triple-panel window with branching wave of $-f$.

#### Proof

The proof is almost verbatim the same as that of Theorem 4.2. We thus restrict ourselves to discussing what happens at the branching points as we monitor the sublevel set of *f* while *t* moves from $-\infty$ to $\infty$. A connected component is born at a minimum or a $\nearrow$-type vertex, *a*, and it dies at a maximum or a $\lambda$-type vertex, *b*. There is neither a birth nor a death when *t* passes the value of a $\searrow$-type vertex or a *y*-type vertex.

Assume *b* is a $\lambda$-type vertex. For $A< t < B$, we have $a \in f_t$ and $b \not \in f_t$. At $t = B$, the component of the sublevel set that contains *a* merges with another, older component and therefore dies. Indeed, this other component approaches *b* from the other branch leading up to *b*, and after *t* passes *B*, the component extends along the branch leaving *b* upwards. $\square$

Note that every vertex is paired only once: the $\nearrow$-type and $\lambda$-type vertices in Phase One, and the $\searrow$-type and y-type vertices in Phase Two. This is in contrast to the critical points in the interior of the edges, which are paired twice. Indeed, according to Definition 4.3, ${W}{({a},{b})}$ is not a window of *f* if *a* is a y-type vertex or *b* is a $\searrow$-type vertex. Symmetrically, ${W}{({b},{a})}$ is not a window of $-f$ if *b* is a $\lambda$-type vertex or *a* is a $\nearrow$-type vertex. In addition to the points in the ordinary and relative subdiagrams—which are characterized by Theorem 4.4—$\textsf{Dgm}_{}{({f})}$ contains one point representing the one component, which is the entire geometric tree, in the essential subdiagram.

### Consequences: symmetry and variation

For a map, *f*, on a geometric tree, the upside-down version of a window of *f* is not necessarily a window of $-f$. The strong symmetry statement in Corollary 3.4 thus fails to generalize and must be replaced by a weaker statement of symmetry. Recall that $\textsf{Dgm}^\circ _{}{({f})}$ and $\textsf{Dgm}^r_{}{({f})}$ are the reflections of $\textsf{Dgm}_{}{({f})}$ through the origin and across the minor diagonal.

#### Corollary 4.5

(Weak Symmetry for Geometric Trees) Let $f :{{\mathbb {A}}}\rightarrow {{\mathbb {R}}}$ be a generic map on a compact geometric tree. Then $\textsf{Dgm}_{}{({-f})} = \textsf{Ord}^\circ _{}{({f})} \sqcup \textsf{Rel}^\circ _{}{({f})} \sqcup \textsf{Ess}^r_{}{({f})}$.

#### Proof

Recall that $\textsf{Dgm}_{}{({f})} = \textsf{Ord}_{}{({f})} \sqcup \textsf{Rel}_{}{({f})} \sqcup \textsf{Ess}_{}{({f})}$. By Theorem 4.4, the triple-panel windows with branching wave of *f* characterize $\textsf{Ord}_{}{({f})}$, and those of $-f$ characterize $\textsf{Rel}_{}{({f})}$. For $-f$, we turn all windows upside-down, which switches and negates coordinates as well as switches the phases in which the windows are constructed. Hence, $\textsf{Ord}_{}{({-f})} = \textsf{Rel}^\circ _{}{({f})}$ and $\textsf{Rel}_{}{({-f})} =\textsf{Ord}^\circ _{}{({f})}$. There is only one point $(A_0, B_0) \in \textsf{Ess}_{}{({f})}$, in which $A_0$ and $B_0$ are the values of the global minimum and the global maximum of *f*. Similarly $\textsf{Ess}_{}{({-f})}$ consists of a single point, $(-B_0, -A_0)$, which completes the proof. $\square$

In contrast, Corollary 3.5 does generalize to geometric trees. However, the windows with non-simple wave complicate the proof of this generalization.

#### Corollary 4.6

(Variation for Geometric Trees) Let $f :{{\mathbb {A}}}\rightarrow {{\mathbb {R}}}$ be a generic map on a geometric tree. Then the variation equals the total persistence: $\textrm{Var}{({f})} = {{\Vert {\textsf{Dgm}_{}{({f})}}\Vert }_1}$.

#### Proof

To formulate the proof strategy, we interpret each point $(A,B) \in \textsf{Dgm}_{}{({f})}$ as the interval with endpoints *A* and *B* on the real line. We will show that for each non-critical value, $t \in {{\mathbb {R}}}$, the cardinality of $f^{-1} (t)$ is equal to the number of intervals in $\textsf{Dgm}_{}{({f})}$ that contain *t*. The claimed equation follows.

To begin, we add every minimum and maximum of *f* as a vertex to ${{\mathbb {A}}}$, so that *f* is monotonic on every edge of the thus subdivided geometric tree. We have six types of vertices, two each of degree 1, 2, and 3. We are interested in the change of the sublevel set and the superlevel set when *t* passes the value of a vertex:

- $\nearrow$-type endpoint: a component of $f_t$ is born;
- $\searrow$-type endpoint: a cycle of $({{\mathbb {A}}}, f^t)$ is born, unless the endpoint is the global maximum, in which case a component of $f_t$ dies.
- minimum: a component of $f_t$ is born and a cycle of $({{\mathbb {A}}}, f^t)$ dies;
- maximum: a component of $f_t$ dies, and a cycle of $({{\mathbb {A}}}, f^t)$ is born, unless the maximum is the global maximum, in which case another component of $f_t$ dies;
- y-type vertex: a cycle of $({{\mathbb {A}}}, f^t)$ dies;
- $\lambda$-type vertex: a component of $f_t$ dies.

We now increase *t* from $- \infty$ to $\infty$. The births and deaths of components correspond to start- and end-points of intervals, while the births and deaths of cycles correspond to end- and start-points of intervals, respectively. Accordingly, the number of intervals that contain *t* increases by 1 when *t* passes the value of a $\nearrow$-type endpoint or a y-type vertex, it decreases by 1 when *t* passes a $\searrow$-type endpoint or a $\lambda$-type vertex, it increases by 2 when *t* passes a minimum, and it decreases by 2 when *t* passes a maximum. The induction basis is provided by *t* smaller than the value of the global minimum, when there are no intervals that contain *t* and there are no points in $f^{-1} (t)$. The induction step is the observation that ${{\#}{f^{-1} (t)}}$ changes in the same way as the number of intervals that contain *t*, namely ${{\#}{f^{-1} (t)}}$ increases by 1 when *t* passes the value of a $\nearrow$-type endpoint or a y-vertex, etc. $\square$

## The general geometric network case

In this section, we take the step from maps on the unit circle and on geometric trees to maps on more general geometric networks. In contrast to a geometric tree, we do not assume that a geometric network is connected.

### Stable marriage

We call an element of $\textsf{H}_1({{\mathbb {G}}})$ a *cycle*, which by definition is an even degree and not necessarily connected subgraph of the network. We relate the global minima and maxima of the cycles in ${{\mathbb {G}}}$ to each other using the notion of a stable marriage. Let $f :{{\mathbb {G}}}\rightarrow {{\mathbb {R}}}$ be a generic map on a compact geometric network, and write $k = {\mathrm{rank\,}{\textsf{H}_1({{\mathbb {G}}})}}$ for the rank of the cycle space. For $\Lambda \in \textsf{H}_1({{\mathbb {G}}})$, we introduce notation for the global minimum and maximum of *f* along $\Lambda$:

3 $$\begin{aligned} {\textrm{lo}{({\Lambda })}}&= \arg \min \nolimits _{x \in \Lambda } f(x) , \end{aligned}$$

4 $$\begin{aligned} {\textrm{hi}{({\Lambda })}}&= \arg \max \nolimits _{x \in \Lambda } f(x) , \end{aligned}$$

calling them the *low point* and the *high point* of the cycle. If cycles $\Lambda \ne \Lambda '$ have the same low point, then genericity implies the existence of a common arc that contains the shared low point in its interior. This arc does not belong to the sum, hence $f({\textrm{lo}{({\Lambda +\Lambda '})}}) > f({\textrm{lo}{({\Lambda })}}) =f({\textrm{lo}{({\Lambda '})}})$. The symmetric inequality holds for cycles with shared high point. Write ${\textrm{Lo}{({f})}}$ and ${\textrm{Hi}{({f})}}$ for the collections of low and high points of all cycles. We begin by proving that both collections have cardinality *k*.

#### Lemma 5.1

(Low and High Points) Let $f :{{\mathbb {G}}}\rightarrow {{\mathbb {R}}}$ be a generic map on a compact geometric network. Then ${{\#}{{\textrm{Lo}{({f})}}}} = {{\#}{{\textrm{Hi}{({f})}}}} ={\mathrm{rank\,}{\textsf{H}_1({{\mathbb {G}}})}}$.

#### Proof

It suffices to prove that ${{\#}{{\textrm{Lo}{({f})}}}}$ is equal to $k={\mathrm{rank\,}{\textsf{H}_1({{\mathbb {G}}})}}$ as the other equality is symmetric. Since $\textsf{H}_1({{\mathbb {G}}})$ is a vector space, every one of its bases consists of *k* cycles. Let $\Lambda _1, \Lambda _2, \ldots , \Lambda _k$ be a basis that maximizes $\sum _{i=1}^k f({\textrm{lo}{({\Lambda _i})}})$. We claim that their low points are distinct. Indeed, if ${\textrm{lo}{({\Lambda _i})}} = {\textrm{lo}{({\Lambda _j})}}$ with $i \ne j$, then $f({\textrm{lo}{({\Lambda _i+\Lambda _j})}}) > f({\textrm{lo}{({\Lambda _j})}})$ and we can substitute $\Lambda _i+\Lambda _j$ for $\Lambda _j$ to get a new basis with larger sum of values. This contradiction implies ${\textrm{lo}{({\Lambda _i})}} \ne {\textrm{lo}{({\Lambda _j})}}$ whenever $i \ne j$ and therefore ${{\#}{{\textrm{Lo}{({f})}}}} \ge k$.

To get ${{\#}{{\textrm{Lo}{({f})}}}} \le k$, we observe that the low point of a sum of cycles in the basis (with distinct low points) is the lowest low point of these cycles and therefore one of the *k* low points we already observed exist. Thus, ${{\#}{{\textrm{Lo}{({f})}}}} = k$, as claimed. $\square$

Since there are equally many low and high points, we can pair them up. Of particular interest is the solution to a *stable marriage* problem (Gale and Shapley 1962). To formulate it, we call $b \in {\textrm{Hi}{({f})}}$ a *candidate* of $a \in {\textrm{Lo}{({f})}}$, and vice versa, if there exists a cycle, $\Lambda$, with $a = {\textrm{lo}{({\Lambda })}}$ and $b={\textrm{hi}{({\Lambda })}}$. Among its candidates, a low point prefers high points with small function values, and a high point prefers low points with large function values. We write ${\textrm{hi}{({a})}}$ and ${\textrm{lo}{({b})}}$ for the *favorites* among their candidates and claim that everybody can be paired with its favorite.

#### Lemma 5.2

(Stable Marriage) Let ${\textrm{Lo}{({f})}}$ and ${\textrm{Hi}{({f})}}$ be the low and high points of a generic map on a compact geometric network, $f :{{\mathbb {G}}}\rightarrow {{\mathbb {R}}}$. Then $\mu :{\textrm{Lo}{({f})}} \rightarrow {\textrm{Hi}{({f})}}$ defined by $\mu (a) = {\textrm{hi}{({a})}}$ is a bijection, and it satisfies $\mu ^{-1} (b) = {\textrm{lo}{({b})}}$.

#### Proof

We show $b = {\textrm{hi}{({a})}}$ iff $a = {\textrm{lo}{({b})}}$, for all $a \in {\textrm{Lo}{({f})}}$ and $b \in {\textrm{Hi}{({f})}}$, which implies the claim. To reach a contradiction, suppose $b = {\textrm{hi}{({a})}}$ but $a' = {\textrm{lo}{({b})}}$ with $a' \ne a$. By definition of favorite, there exists a cycle, $\Lambda$, with ${\textrm{lo}{({\Lambda })}} = a$ and ${\textrm{hi}{({\Lambda })}} = b$. Hence, *a* is a candidate of *b*. However, since $a' \ne a$ is the favorite of *b*, this implies $f(a') > f(a)$. Let $\Lambda '$ be the cycle with ${\textrm{lo}{({\Lambda '})}} = a'$ and ${\textrm{hi}{({\Lambda '})}} = b$. Then ${\textrm{lo}{({\Lambda +\Lambda '})}} = a$ and $f({\textrm{hi}{({\Lambda +\Lambda '})}}) < f(b)$, which contradicts that *b* is the favorite of *a*. $\square$

### Maps on geometric networks

The components and cycles of ${{\mathbb {G}}}$ give rise to points in the 0- and 1-dimensional essential subdiagrams of $\textsf{Dgm}_{}{({f})}$. They need new kinds of windows to be recognized. The more interesting case is that of a cycle. Let $a \in {\textrm{Lo}{({f})}}$, $b \in {\textrm{Hi}{({f})}}$, and recall the definition of $J = {J}{({a},{b})}$. If *a* and *b* are candidates of each other, then $J \ne \emptyset$ as it contains at least the cycles whose low and high points are *a* and *b*. Even if *a* and *b* are not candidates of each other, $J \ne \emptyset$ is possible, but then it does not contain any cycle through the two points.

#### Definition 5.3

(Windows for Geometric Networks) Let $a \in {{\mathbb {G}}}$ be a minimum, $\nearrow$-type, or y-type vertex, with $f(a) = A$, and $b \in {{\mathbb {G}}}$ a maximum, $\searrow$-type, or $\lambda$-type vertex, with $f(b) = B$. Recall that $J = {J}{({a},{b})}$ is the component of $f^{-1} [A,B]$ that contains both *a* and *b*, with $J = \emptyset$ if no such component exists.

(i): ${W}{({a},{b})} = J \times [A,B]$ is a *window of component* if *J* is an entire component of ${{\mathbb {G}}}$.
(ii): ${W}{({a},{b})}$ is a *window of cycle* if *J* contains a cycle that passes through *a* and *b* such that $J \setminus \{a, b\}$ is not connected.

**Fig. 5:**
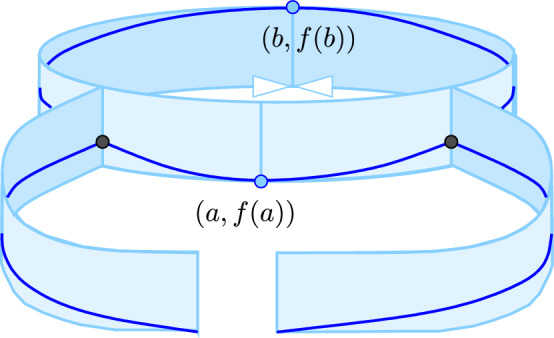
A window of cycle. If the two arms met at the ends, this would be a violation of the conditions in Definition 5.3(ii) since cutting at *a* and *b* would not disconnect the strip

The window of cycle is illustrated in Fig. 5: (*a*, *A*) and (*b*, *B*) lie on the lower and upper boundaries of the cylindrical strip. If ${W}{({a},{b})}$ does not satisfy the conditions in Definition 5.3(ii), then cutting the strip along vertical lines at *a* and *b* does not split it into two connected pieces. On the other hand, if ${W}{({a},{b})}$ is a window of cycle, then the two cuts split the strip into two components. Note that a window with wave can neither be a window of component nor of cycle. On the other hand, it is possible that a window of component is also a window of cycle.

The proof of Lemma 5.2 implies that ${W}{({a},{b})}$ is a window of cycle iff *a* and *b* are each other’s favorites. We show that this is also equivalent to being paired in persistent homology.

#### Theorem 5.4

(Characterization for Compact Geometric Networks) Let $f :{{\mathbb {G}}}\rightarrow {{\mathbb {R}}}$ be a generic map on a compact geometric network, let *a* be a minimum, $\nearrow$-type, or y-type vertex, with $A = f(a)$, and let *b* be a maximum, $\searrow$-type, or $\lambda$-type vertex, with $B = f(b)$. Then

(i): $(A,B) \in \textsf{Ess}_{0}{({f})}$ iff ${W}{({a},{b})}$ is a window of component,
(ii): $(B,A) \in \textsf{Ess}_{1}{({f})}$ iff ${W}{({a},{b})}$ is a window of cycle.

#### Proof

(i) is obvious enough so we omit the proof. To see (ii), assume *a* and *b* are each other’s favorites, and let $\Lambda$ be a cycle whose low and high points are *a* and *b*. When $t \in {{\mathbb {R}}}$ reaches *B* in Phase One, $\Lambda$ is born along with all cycles $\Lambda + \Lambda '$, in which $\Lambda '$ is a cycle born before $\Lambda$. All these cycles die when *t* reaches *A* in Phase Two. Indeed, if $\Lambda '$ died earlier, then $\Lambda + \Lambda '$ would become homologous to $\Lambda$, but since $\Lambda$ is born after $\Lambda '$, the sum of the two cycles cannot die yet. On the other hand, $\Lambda + \Lambda '$ dies at $t = A$ because it becomes homologous to $\Lambda '$, which was born earlier. $\square$

The characterization of points in the essential subdiagram of $\textsf{Dgm}_{}{({f})}$ in Theorem 5.4 together with the characterization of the points in the ordinary and relative subdiagrams in Theorem 4.4 completes the proof of the Main Theorem stated in the Introduction.

### Consequences: symmetry and variation

The weak symmetry assertion for geometric trees stated in Corollary 4.5 generalizes to geometric networks.

#### Corollary 5.5

(Weak Symmetry for Geometric Networks) Let $f :{{\mathbb {G}}}\rightarrow {{\mathbb {R}}}$ be a generic map on a compact geometric network. Then $\textsf{Dgm}_{}{({-f})} = \textsf{Ord}^\circ _{}{({f})} \sqcup \textsf{Rel}^\circ _{}{({f})} \sqcup \textsf{Ess}^r_{}{({f})}$.

#### Proof

The argument for the triple-panel windows with wave is the same as in the proof of Corollary 4.5. Since geometric networks are not necessarily connected, we can have more than one window of component, which is different for geometric trees, which are connected. Nevertheless, the argument for such windows is the same as in the proof of Corollary 4.5.

It remains to argue about the cycles in the network. By Lemma 5.2, the cycles are represented by pairing their low and high points in a symmetric manner. Specifically, each low point is paired with the lowest candidate high point, and because the candidate relation is symmetric, this is equivalent to pairing each high point with the highest candidate low point. Each such pair generated in Phase One corresponds to a point $(A,B) \in \textsf{Ess}_{}{({f})}$, and by symmetry to a point $(-B,-A) \in \textsf{Ess}_{}{({-f})}$, which completes the proof. $\square$

The equality of the variation and the total persistence generalizes from circles and geometric trees to geometric networks. We can reuse the proof of Corollary 4.6, which we complement with an argument about cycles.

#### Corollary 5.6

(Variation for Geometric Networks) Let $f :{{\mathbb {G}}}\rightarrow {{\mathbb {R}}}$ be a generic map on a compact geometric network. Then the variation is the total persistence: $\textrm{Var}{({f})} = {{\Vert {\textsf{Dgm}_{}{({f})}}\Vert }_1}$.

#### Proof

We cut each cycle in ${{\mathbb {G}}}$ at its high point to obtain a geometric network, ${{\mathbb {G}}}'$, with one less cycle. Let $\eta :{{\mathbb {G}}}' \rightarrow {{\mathbb {G}}}$ be the surjection that reverses the cut, and let $g :{{\mathbb {G}}}' \rightarrow {{\mathbb {R}}}$ be defined by $g(x) = f(\eta (x))$. Since the maps are essentially the same, we have $\textrm{Var}{({g})} = \textrm{Var}{({f})}$.

To show that the total persistence remains the same, let $\Lambda$ be a cycle in ${{\mathbb {G}}}$, $a = {\textrm{lo}{({\Lambda })}}$ its low point, and $b={\textrm{hi}{({\Lambda })}}$ its high point. Assume that ${W}{({a},{b})}$ is a window of cycle, so that $(A,B) \in \textsf{Ess}_{1}{({f})}$, in which $A = f(a)$ and $B = f(b)$, as usual. The cut at *b* removes the cycle and thus the point (*A*, *B*) from the diagram. There is a second window, generated by *b* and another point $x \in {{\mathbb {G}}}$, whose corresponding point, (*B*, *X*), is removed from the diagram. In lieu of *b*, we get two $\nearrow$-type endpoints in ${{\mathbb {G}}}'$, which we denote $b'$ and $b''$. By definition of $\eta$, we have $g(b') = g(b'') = B$. Since $b'$ and $b''$ are endpoints, they are paired only once. By the local characterization of windows in Theorems 3.2, 4.2, 4.4, 5.4, all windows of *f* other than ${W}{({a},{b})}$ and ${W}{({b},{x})}$ are also windows of *g*. Hence $b'$ and $b''$ can only be paired with *a* and *x*. We thus get two new points, (*B*, *A*) and (*B*, *X*) in $\textsf{Dgm}_{}{({g})}$. Their persistence is the same as that of the two points they replace, so ${{\Vert {\textsf{Dgm}_{}{({g})}}\Vert }_1} = {{\Vert {\textsf{Dgm}_{}{({f})}}\Vert }_1}$.

We now repeat the argument, cutting one cycle at the time, until we reach a collection of geometric trees. Corollary 4.6 implies that the variation is equal to the total persistence. Since both quantities did not change during the process, we thus established the equality also for compact geometric networks. $\square$

## Discussion

The main contribution of this paper is the local characterization of points in the extended persistence diagram of a map on a compact geometric network. This work gives rise to a number of open questions, of which we state two:

- The characterization through critical point pairs by windows identifies endpoints and branching points as culprits for the failure of $\textsf{Dgm}_{}{({f})} = \textsf{Dgm}^R_{}{({f})}$ beyond circles. Can we sharpen this to a quantitative relationship between the symmetric difference of the two diagrams and the number of endpoints and branching points in the geometric network?
- While the variation is a natural concept for 1-dimensional maps, there are several competing extensions to maps on 2- and higher-dimensional domains (Hardy–Wright variation, Harman variation, etc.); see e.g. Pausinger and Svane (2015). How does the total persistence of such a map relate to these extensions?

In conclusion, we mention the development of a fast dynamic data structure for maintaining the persistence diagram of lists (Cultrera di Montesano et al. 2023), which is based on the results of this paper. It paves the way to efficient software for the persistence of long time-series. Are there questions for series with bifurcations (modeled by geometric trees and networks) that benefit from the ready availability of the persistence diagram?
